# Emergence and spread of NA-I223V and NA-S247N double-mutant A(H1N1)pdm09 influenza viruses with reduced oseltamivir susceptibility in the Netherlands and beyond, 2023 to 2026

**DOI:** 10.2807/1560-7917.ES.2026.31.36.2600733

**Published:** 2026-09-10

**Authors:** Zandra Felix Garza, Dirk Eggink, Mariam Bagheri, Sharon van den Brink, Gabriel Goderski, Mark Pronk, Pascal Lexmond, Mariëtte Hooiveld, Rianne van Gageldonk-Lafeber, Björn Koel, Ron Fouchier, Adam Meijer

**Affiliations:** 1 Centre for Infectious Disease Control, National Institute for Public Health and the Environment, Bilthoven, The Netherlands; 2 Department of Viroscience, Erasmus Medical Centre, Rotterdam, The Netherlands; 3 Nivel (Netherlands institute for health services research), Utrecht, The Netherlands

**Keywords:** A(H1N1)pdm09 influenza virus, antiviral susceptibility, oseltamivir, zanamivir, phenotypic, genomic

## Abstract

In 2023/24, A(H1N1)pdm09 influenza viruses with neuraminidase (NA)-S247N emerged in NA-clade C.5.3.3 carrying NA-I223V, spread internationally, then faded. Such double mutants reappeared sporadically in 2024/25. They expanded again in 2025/26 in NA-clade D.3 viruses carrying NA-S247N after acquiring NA-I223V in Europe – notably Spain, the Netherlands, Finland and France, and beyond. Dutch double mutants from both seasons showed median 12- and 13-fold reduced inhibition by oseltamivir. These findings underscore the need for ongoing genomic and phenotypic monitoring of antiviral susceptibility.

In 2023/24, a neuraminidase (NA)-clade of A(H1N1)pdm09 influenza viruses carrying the NA-I223V amino-acid substitution emerged, followed by acquisition of NA-S247N [1-3]. Both substitutions individually increase the 50 per cent inhibitory concentration (IC_50_) by oseltamivir but the resulting IC_50_-fold-increase remains below the threshold for reduced inhibition (RI) (IC_50_-fold-change > 10 compared with median IC_50_ of wildtype (WT) viruses [4]). In double-mutant viruses, the substitutions act synergistically, causing phenotypically RI by oseltamivir, but not by zanamivir [1,2]. In late 2025, Saubi et al. [5] reported re-emergence of A(H1N1)pdm09 viruses carrying NA-S247N in Spain and elsewhere in Europe. Here, we show the re-emergence of A(H1N1)pdm09 NA-I223V and NA-S247N double-mutant viruses in 2025/26 in the Netherlands and assess their phenotypic susceptibility, global spread and evolution from 2023 to 2026.

## Neuraminidase genomic assessment, the Netherlands

Dutch A(H1N1)pdm09 viruses were collected between October 2023 and April 2026 from three surveillance systems focusing on different levels of disease severity: (i) the syndromic surveillance system ‘Infectieradar’ where community-dwelling individuals participating with self-reported acute respiratory infection (ARI), submit self-collected combined nose and oropharyngeal swab specimens; (ii) the sentinel general practitioners (GPs) collecting combined nasopharyngeal and oropharyngeal swab specimens from patients diagnosed with ARI; (iii) a network of ca 24 clinical diagnostic laboratories sending a subset of influenza virus positive clinical specimens to the National Influenza Centre (NIC) [6-8]. Sequences of 2,258 A(H1N1)pdm09 viruses, obtained with next-generation nanopore sequencing [9], were used for haemagglutinin (HA) and NA-clade assignment using Nextclade v3.21.2 (https://clades.nextstrain.org) [10]. 

**Figure 1:**
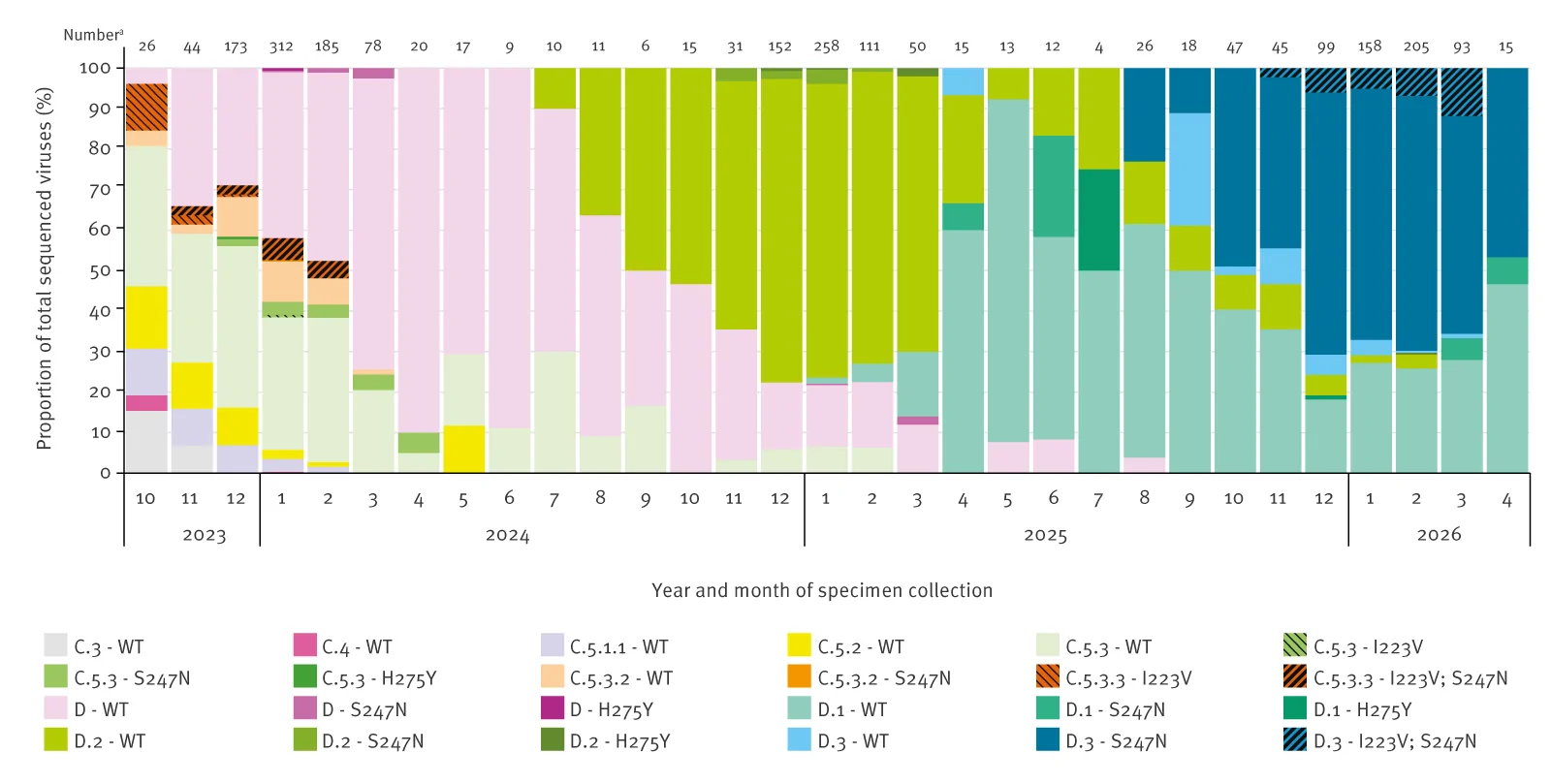
Neuraminidase (NA)-clade distribution over time of A(H1N1)pdm09 influenza viruses without and with amino-acid substitutions affecting antiviral susceptibility available by 23 May 2026 with specimen collection dates in October 2023 through April 2026, the Netherlands (n = 2,258)

In 2023/24, the NAs of A(H1N1)pdm09 belonged to eight different NA-clades (Figure 1). In NA-clade C.5.3, NA-I223V became fixated in NA-subclade C.5.3.3. Within C.5.3.3, a cluster of viruses emerged that also carried NA-S247N. Of the eight clades, six faded by March 2024 and two continued fading. Viruses belonging to subsequent dominant NA-clades (in the order of D, D.2, D.1) sporadically displayed NA-S247N, D.1 more extensively than the other clades (Figure 1). During the 2024/25 season, both substitutions were only sporadically observed. However, in NA-clade D.3 viruses that already carried NA-S247N from the start of spreading in August 2025, NA-I223V emerged in a defined subcluster in November 2025. The proportion of double mutants reached a maximum in March 2026 after which NA-clade D.3 viruses started to fade. By then, NA-S247N had already re-emerged in NA-clade D.1 and NA-clade D.1 viruses proportionally increased in April among detected A(H1N1)pdm09 viruses (Figure 1).

IC_50_: 50% inhibitory concentration; NA: neuraminidase; WT: wildtype.

^a^ Number of A(H1N1)pdm09 viruses characterised each month.

Reduced inhibition by oseltamivir is defined as an IC_50_ 10 to 100-fold above WT median IC_50_; highly reduced inhibition is defined as an IC_50_ > 100-fold above WT median IC_50_. Each clade in the graph is indicated by a distinct colour with three possible shades including light, dark and intermediary. The light shade indicates the WT. The dark shade indicates the presence of the NA-H275Y amino-acid substitution, which is associated with highly reduced inhibition by oseltamivir. The intermediary shade indicates (i) either double mutations leading to reduced inhibition (NA-I223V + NA-S247N; with thick upward striped background) or (ii) single mutations associated with higher oseltamivir IC_50_ values but below the fold-change threshold for reduced inhibition (NA-S247N with plain background or NA-I223V with thin downward striped background).

## Phenotypic assessment, the Netherlands

A subset of 182 Dutch A(H1N1)pdm09 viruses from the three seasons, were selected based on NA sequence analysis of published antiviral marker positions [11]. Both antiviral-marker-position WT viruses and mutant viruses were included in this subset. The selected viruses were isolated and phenotypically characterised by MUNANA-based NA enzyme inhibition assay for sensitivity to oseltamivir and zanamivir [12]. The World Health Organization (WHO) recommended approach to calculate IC_50_-fold-changes is using the season matched WT viruses IC_50_ data [4,11]. This approach has limitations, particularly in 2025/26 when WT A(H1N1)pdm09 viruses showed slightly higher oseltamivir IC_50_ values (median: 0.86 nM; 16 WT viruses among 50 analysed) than in 2023/24 (0.57 nM; 61 WT/87 analysed) and 2024/25 (0.50 nM; 40 WT/47 analysed), despite lacking known RI-associated amino-acid substitutions in the NA. This shift was not observed for 2025/26 WT A(H3N2) viruses (median IC_50_ for oseltamivir respectively 0.27 nM (n = 63 viruses analysed), 0.24 nM (n = 44) and 0.21 nM (n = 88), indicating it was not reagent-related and likely inherent to the NA backbone of 2025/26 A(H1N1)pdm09 viruses. Using the 2025/26 WT median, 2025/26 double-mutant viruses showed 7.9–10.2-fold increases for oseltamivir, just crossing the RI-threshold of 10-fold. When using reference WT virus A(H1N1)pdm09 A/Illinois/45/2019 (IRR FRS-1748; median IC_50_ 0.46 nM for oseltamivir; n = 18), oseltamivir IC_50_-fold-changes for 2025/26 double mutants increased to 14.9–19.3-fold.

**Figure 2:**
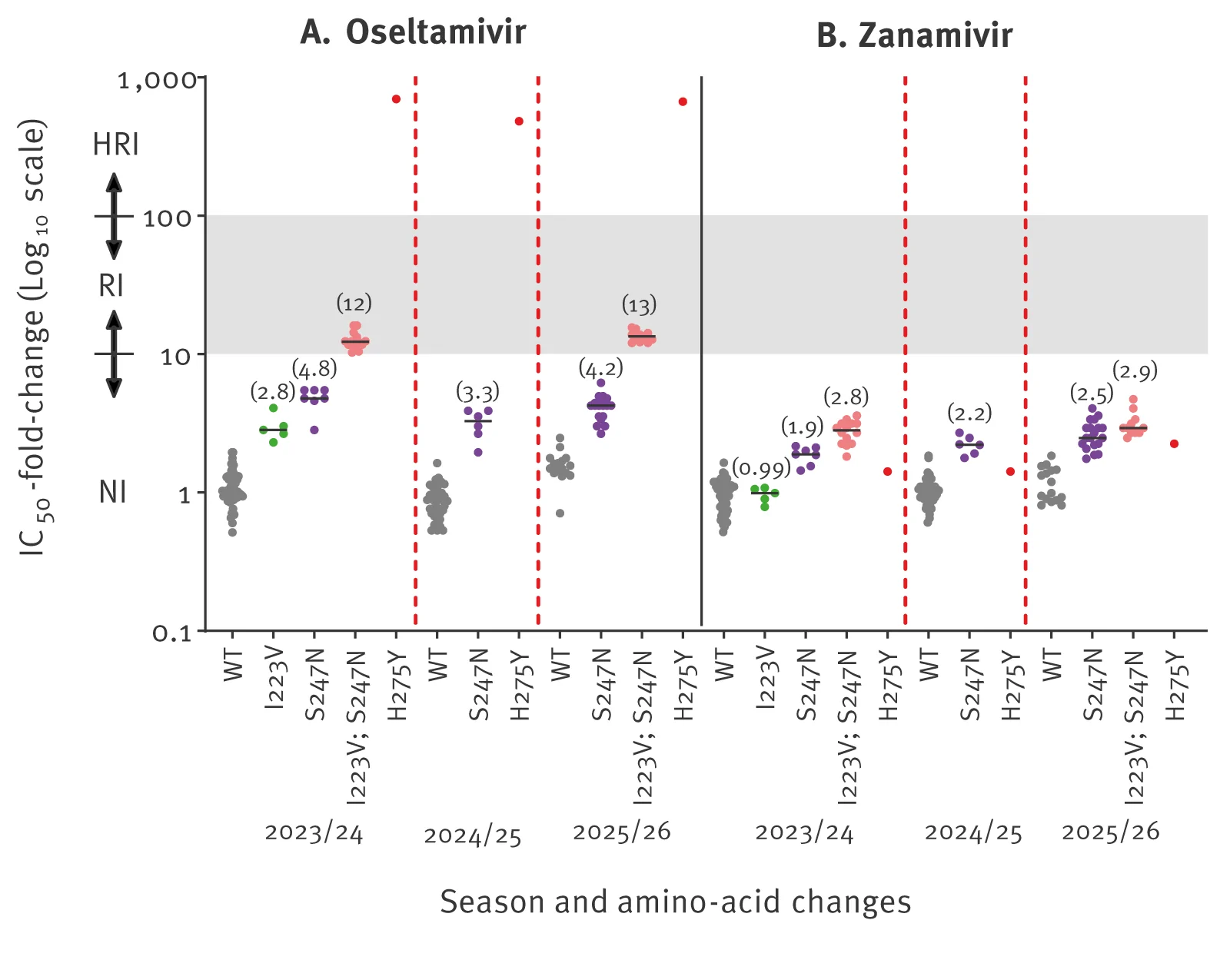
IC_50_-fold-change compared with median of wildtype virus for (A) oseltamivir and (B) zanamivir for isolated and phenotypically analysed wildtype and mutant A(H1N1)pdm09 influenza viruses available by 23 May 2026 with specimen collection dates in October 2023 through April 2026, the Netherlands (n = 182)

To allow consistent comparison of mutant viruses across the analysed seasons, three-season median IC_50_s of WT viruses (0.57 nM for oseltamivir and 0.45 nM for zanamivir; n = 117) were used to calculate fold-changes for all mutant viruses. The single-mutant viruses NA-I223V and NA-S247N already showed a shift in IC_50_-fold-change to higher values for oseltamivir and zanamivir, but with fold-change below the RI-threshold of 10 (Figure 2). The double mutants with both NA-I223V and NA-S247N in the 2023/24 and 2025/26 seasons clearly showed comparable RI by oseltamivir just above the RI-threshold (Figure 2). The synergistic effect was lower for zanamivir, rendering still normal inhibition by zanamivir for these viruses (Figure 2).

IC_50_: 50% inhibitory concentration; HRI: highly reduced inhibition (> 100-fold above wildtype median IC_50_); NA: neuraminidase; NI: normal inhibition (< 10-fold above wildtype median IC_50_); RI: reduced inhibition (10 to 100-fold above wildtype median IC_50_); WT: wildtype.

Detected A(H1N1)pdm09 viruses with NA-H275Y were included as highly reduced inhibition by oseltamivir and normal inhibition by zanamivir references. In brackets and black horizontal overlaid line the median fold-changes of mutants compared with WT median IC_50_.

## Phylogenetic characterisation, the Netherlands

**Figure 3:**
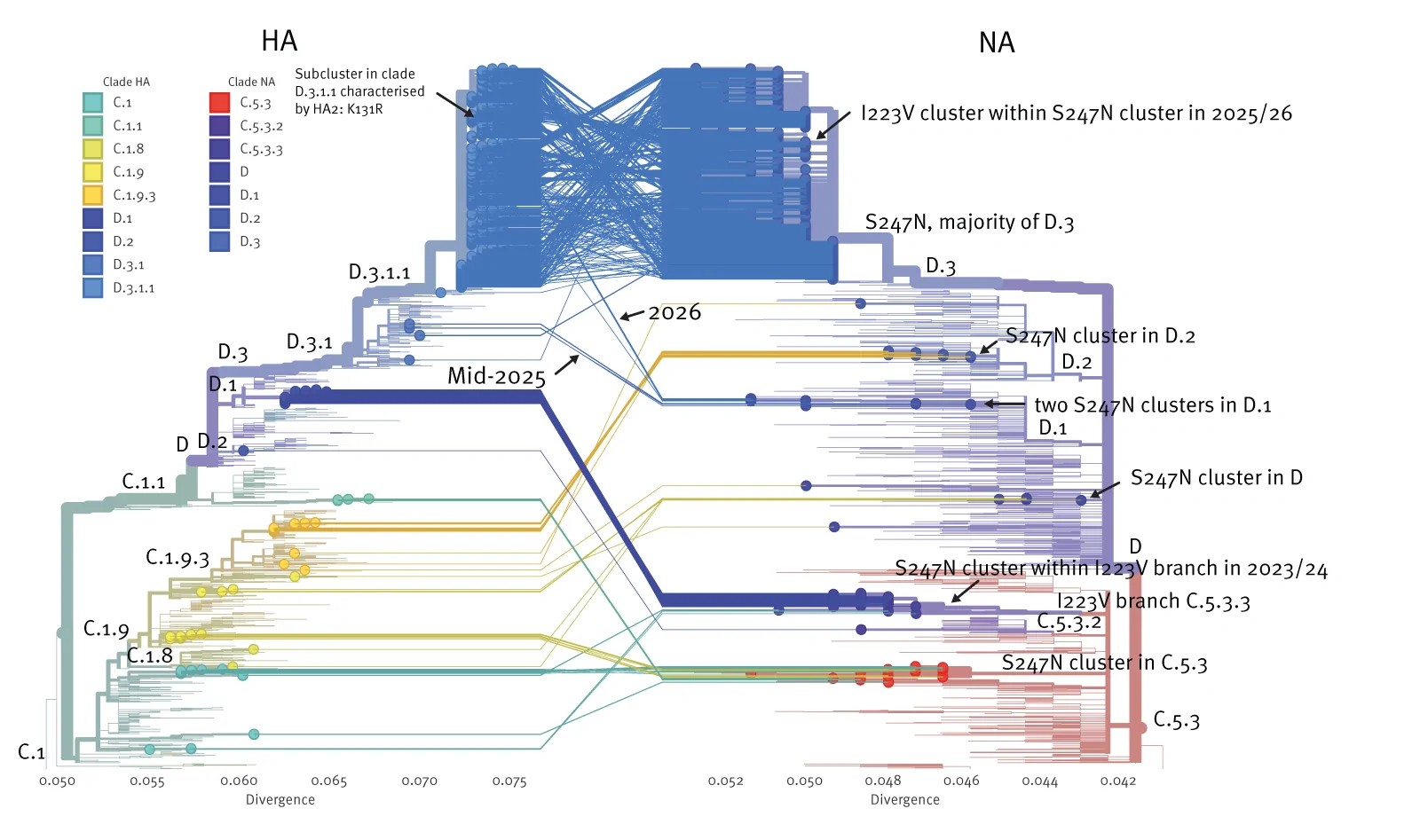
Tanglegram of haemagglutinin (HA) and neuraminidase (NA) sequences of A(H1N1)pdm09 influenza viruses with NA-I223V and/or NA-S247N available by 23 May 2026 with specimen collection dates in October 2023 through April 2026, the Netherlands (n = 526)

HA: haemagglutinin; NA: neuraminidase.

Nucleotide HA and NA sequences of 526 NA-I223V and/or NA-S247N containing Dutch viruses were used to create a tanglegram (Figure 3) to investigate association of NA-clusters characterised by specific antiviral traits with specific HA-clusters. In 2023/24, the double mutants belonged to one NA-subcluster, defined by NA-S247N, within the larger NA-clade C.5.3.3, defined by NA-I223V. Furthermore, NA-clade C.5.3.3 was mainly associated with the HA-clade D.1, that was exclusively associated with the C.5.3.3 NA-S247N subcluster, and occasionally with two different branches of HA-clade C.1. In 2025/26, NA-S247N emerged first and NA-I223V was subsequently acquired. Interestingly, the double mutants were allocated to one subcluster within one NA-clade (D.3), similar to 2023/24. This subcluster was exclusively associated with the subcluster of HA-clade D.3.1.1 defined by HA2-K131R. The upsurge of NA-clade D.1 viruses with NA-S247N in 2026 occurred in a different HA background, HA-clade D.3.1.1 (not the subcluster characterised by HA2-K131R), than in mid-2025, when the NA-clade D.1 viruses with NA-S247N were associated with HA-clade D.3.1.

Dutch viruses (coloured dots) are placed in the context of global A(H1N1)pdm09 viruses derived from Nextclade phylogenetic analysis (lines without dots) of HA and NA sequences individually. The HA sequence and corresponding NA sequence of each virus are connected by lines. Amino-acid changes common to relevant defined clusters are shown at the branches leading to those clusters and specifically highlighted in the NA tree for NA-I223V and NA-S247N. In addition to tip colouring representing individual HA and NA clades, also the branches leading to these clades are highlighted with clade annotation. The tanglegram was constructed using Nextclade phylogeny and visualisation by Auspice v2.73.0 (https://auspice.us/) (HA and NA reference dataset built 2026–03–22 HA:MW626062 and NA:MW626056) [18,19].

## Global neuraminidase genomic assessment

**Figure 4:**
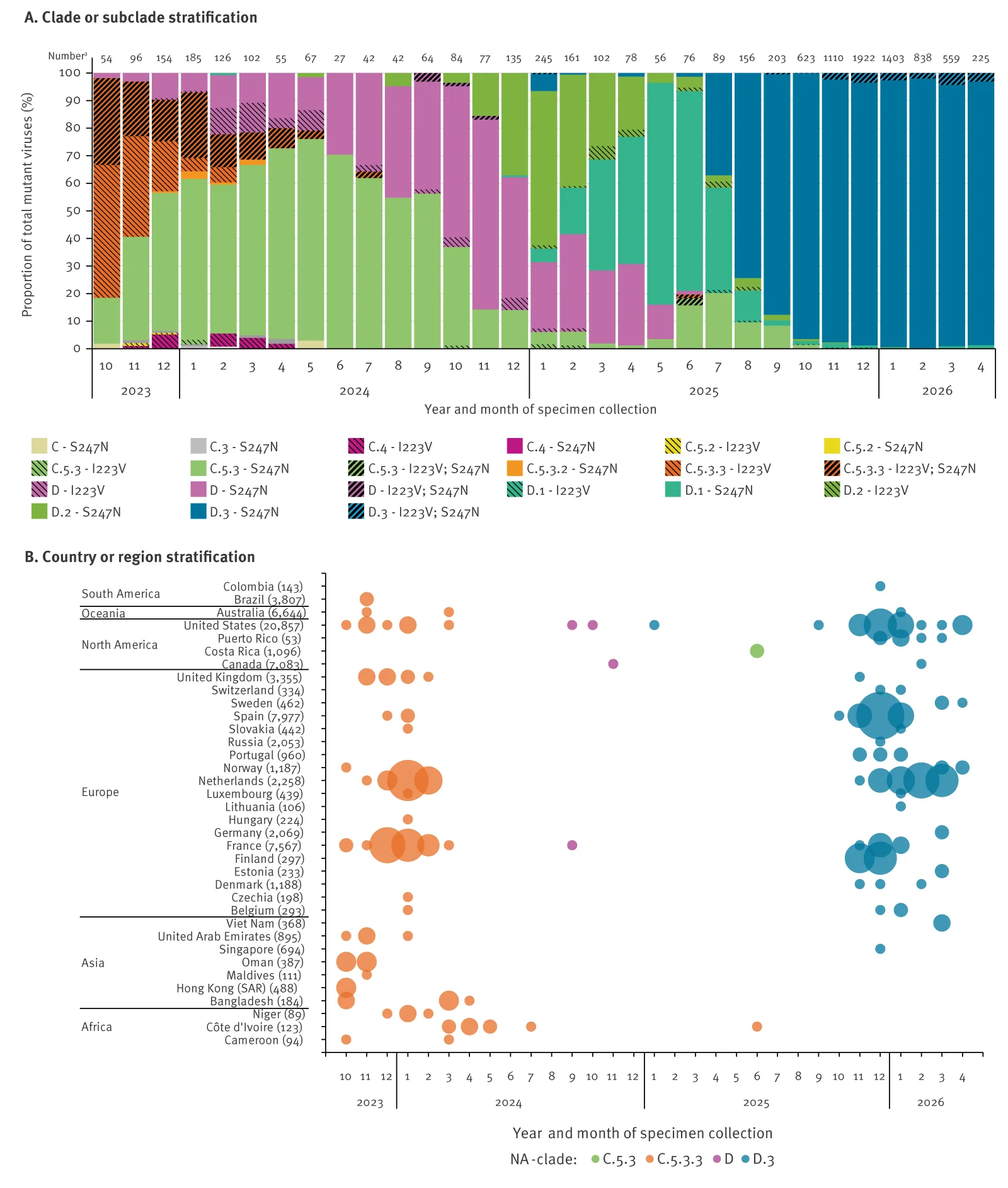
Seasonal analysis stratified by (A) clade/subclade and (B) place, of neuraminidase (NA) sequences of NA-I223V and/or NA-S247N mutant A(H1N1)pdm09 influenza viruses available by 3 August 2026 collected worldwide, between October 2023 and April 2026 (n = 9,156)

^a^ Number of A(H1N1)pdm09 viruses with NA-I223V and/or NA-S247N characterised each month.

For analysis of worldwide detected A(H1N1)pdm09 viruses collected between October 2023 and April 2026, 9,975 NA sequences with NA-I223V and/or NA-S247N were downloaded from GISAID using the mutant selection tool [13]. A total of 9,156 NA sequences remained after removing embargoed, duplicate (same strain name), and low-quality sequences that failed Nextclade NA-clade assignment. The NA-clade distribution of mutant viruses over time was similar to that for the Netherlands (Figure 4a). The majority of double-mutant viruses was observed in Europe (Spain, the Netherlands, Finland and France) and the United States, both in the 2023/24 and 2025/26 seasons (Figure 4b). However, this is partially driven by the total number of sequenced viruses per country. The majority of 2023/24 double mutants were in the same subcluster of NA-clade C.5.3.3 as for the Netherlands, but occurred also in three other branches of NA-clade C.5.3.3, as described in Supplementary Figure S1. Similarly, the majority of 2025/26 double mutants were in the same subcluster of NA-clade D.3 as for the Netherlands, but occasionally occurred also in two other branches of D.3, as illustrated in Supplementary Figure S2. Different from the Netherlands, double mutants were also sporadically found in NA-clades C.5.3 and D (Figure 4a). Furthermore, single mutant NA-I223V viruses worldwide were found in more NA-clades and in a higher frequency than in the Netherlands. Importantly, 34 mutant viruses from across the world carried NA-H275Y associated with oseltamivir highly RI in addition to NA-S247N (n = 33) or NA-I223V + NA-S247N (n = 1).

NA: neuraminidase.

**A.** Proportion distribution by NA-clade over time for the mutant viruses (n = 9,156; 118 countries/territories including Japan). Distinct colour families indicate the clades or subclades while patterns indicate presence of amino-acid changes associated with either higher oseltamivir IC_50_ values (NA-I223V; thin downward striped background or NA-S247N; plain background) but below the fold-change threshold for reduced inhibition, or oseltamivir reduced inhibition (NA-I223V + NA-S247N; with thick upward striped background). Reduced inhibition is defined as an IC_50_ 10 to 100-fold above wildtype median IC_50_. **B.** Global distribution over time of double-mutant NA-I223V + NA-S247N viruses showing reduced inhibition by oseltamivir by region and country (n = 323). Bubble size is proportional to virus count, with the smallest bubble size accounting for one virus and the largest size for 23 viruses. Bubbles are coloured in the same way as in Figure 4a for the NA-clade. Shown in brackets after the country names are the raw total number of A(H1N1)pdm09 viruses for which NA sequences were available in GISAID with a collection date between October 2023 and April 2026.

## Discussion

The recent expansion of A(H1N1)pdm09 influenza virus clusters carrying either NA-I223V (2023/24) or NA-S247N (2025/26) was followed in both seasons by the emergence and spread of double-mutant viruses combining these substitutions, causing RI by oseltamivir. This pattern suggests a fitness advantage of single and double mutants [2], although they have not become permanently dominant. The appearance of double mutants is likely independent of antiviral selection, as NA inhibitors are only sporadically used in several countries reporting high proportions of these viruses and such viruses have not been reported from Japan, where NA inhibitor use by capita is highest [14,15], in publicly available GISAID data.

NA-I223V and NA-S247N, alone or in combination, have been proposed as changes that compensate for the fitness cost of NA-H275Y, which confers highly RI by oseltamivir [16]. Both substitutions individually, and especially together, further drastically reduce inhibition by oseltamivir in NA-H275Y mutants [16,17]. During the study period, NA-S247N together with NA-H275Y was detected 33 times and once as a triple mutant including NA-I223V, confirming that such variants showing very high RI can arise and are of concern. Phenotypic data for Dutch viruses show that the 2025/26 NA-I223V combined with NA-S247N double mutant display oseltamivir RI while retaining normal inhibition by zanamivir, similar to the 2023/24 double-mutant and reports elsewhere [1,2,17]. The shown impact of different reference IC_50_ values on fold-change calculations highlights the need for caution in using WT data and interpreting IC_50_ values near the 10-fold RI threshold, given the arbitrary nature of the defined threshold values [11]. Synergistic IC_50_ increases in double mutants should nonetheless be carefully monitored and reported. The impact of these double mutants on clinical management of influenza patients is unknown and should be part of future studies.

Dominance of double mutants within defined HA/NA-subclusters, with gradual accumulation of additional changes, suggests global dissemination from one or several initial emergence(s) or introduction(s), although occasional detection of double mutants in other NA-clades indicates that parallel evolution of similar A(H1N1)pdm09 variants showing oseltamivir RI is also possible. Awareness is therefore warranted whenever NA-I223V or NA-S247N become fixed in spreading subclusters. The recent emergence and spread of NA-S247N in NA-clade D.1 on an HA-clade D.3.1.1 background should be closely monitored during the progressing 2026 southern hemisphere and coming 2026/27 northern hemisphere seasons. Similar to the abundancy of NA-clade D.3 with NA-S247N in HA-clade D.3.1.1 background, HA-clade D.3.1.1 viruses could provide a favourable context for the expansion of such viruses carrying NA of clade D.1 with NA-S247N.

Our global assessment is limited by reliance on non‑embargoed sequences and metadata in GISAID. Country and regional representativeness depends on local laboratory capacity to sequence at least the HA and NA gene segments, and on whether data are submitted to GISAID or other databases (e.g. GenBank, Pathoplexus). While WHO Collaborating Centres partly compensate for gaps at NICs by sequencing of representative viruses shared by NICs and submitting these sequence data to GISAID, our analysis may still over‑estimate mutant presence in some regions and under‑estimate it in others. Our study outcomes should therefore be interpreted in light of these sampling and database‑related limitations.

## Conclusion

Our findings highlight the need for continued surveillance on the evolution of A(H1N1)pdm09 influenza viruses and the possible emergence of mutants, especially double and triple ones, with antiviral (highly) RI. The capacity to phenotypically evaluate the impact of individual and combinations of amino-acid substitutions associated with antiviral (highly) RI remains a critical component of this surveillance.

## Data Availability

The sequences from the Netherlands and sequences from other countries with associated metadata available on GISAID are accessible at https://doi.org/10.55876/gis8.260806vs (sequences of the Netherlands), https://doi.org/10.55876/gis8.260816ae (sequences of 136 other countries/territories used in the phylogenetic analysis of Dutch sequences) and https://doi.org/10.55876/gis8.260815oq (sequences of the Netherlands and of 117 other countries/territories used for the global phylogenetic analysis).

## Supplementary Data

46000 Supplementary Material
