# Supplementary material for: Emergence and spread of NA-I223V and NA-S247N double-mutant A(H1N1)pdm09 influenza viruses with reduced oseltamivir susceptibility in the Netherlands and beyond, 2023 to 2026
Source: Euro Surveill. 2026 Sep 10;31(36):2600733. doi: 10.2807/1560-7917.ES.2026.31.36.2600733 (PMC13570233; doi:10.2807/1560-7917.ES.2026.31.36.2600733)
Supplement: Supplementary file 1 [file 26-00733_MEIJER_Supplementary_material.pdf]

Supplementary material

This supplementary material is hosted by Eurosurveillance as supporting information alongside the article *Emergence and spread of NA-I223V and NA-S247N double-mutant A(H1N1)pdm09 influenza viruses with reduced oseltamivir susceptibility in the Netherlands and beyond, 2023-2026*, on behalf of the authors, who remain responsible for the accuracy and appropriateness of the content. The same standards for ethics, copyright, attributions and permissions as for the article apply. Supplements are not edited by Eurosurveillance and the journal is not responsible for the maintenance of any links or email addresses provided therein.

Supplementary Figure S1. Phylogenetic analysis of neuraminidase(NA) sequences of NA-I223V and/or NA-S247N mutant A(H1N1)pdm09 influenza viruses available by 3 August 2026 collected worldwide between October 2023 and April 2026

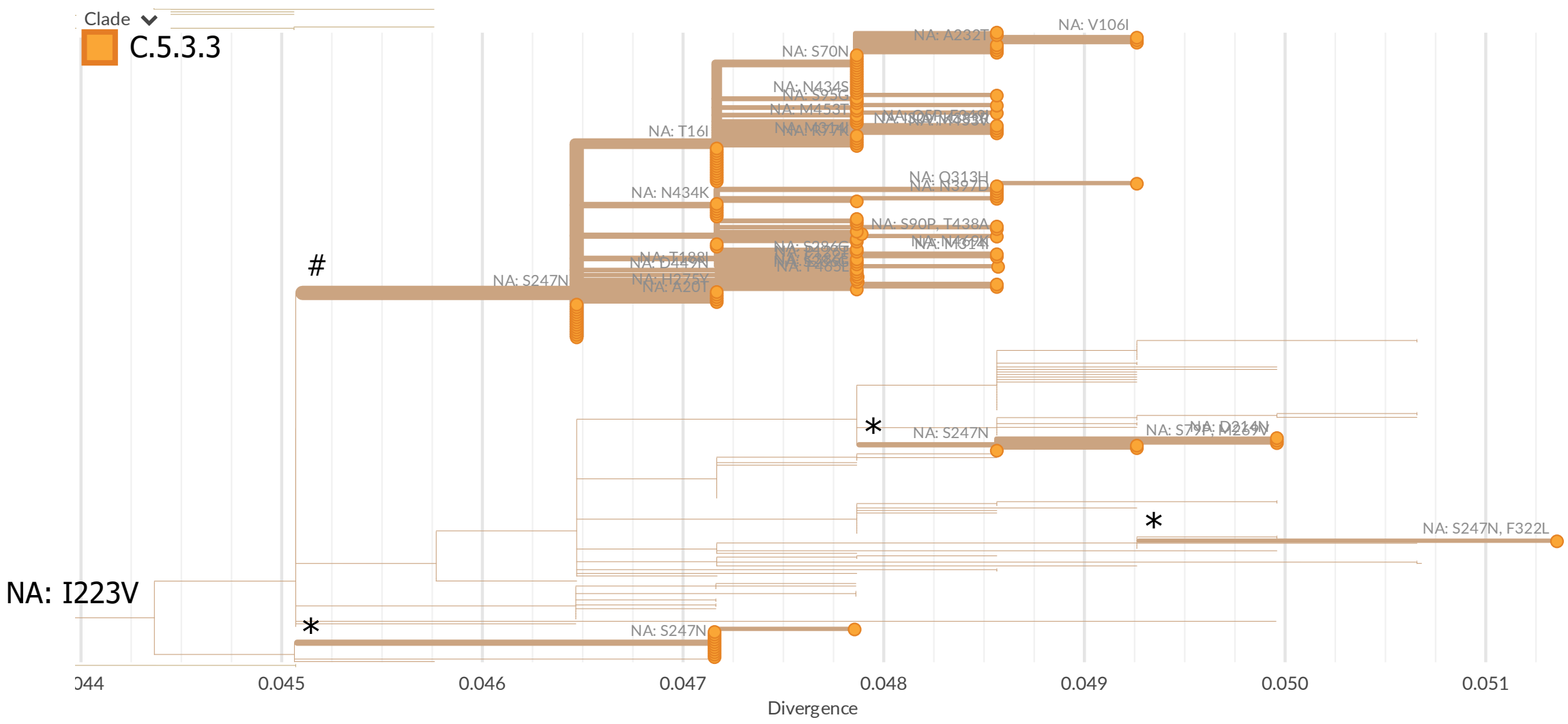

Viruses with NA-I223V and NA-S247N (coloured dots) are placed in the context of viruses that carry NA-I223V or NA-S247N and reference viruses (lines without dots) derived from Nextclade phylogenetic analysis. The NA-clade C.5.3.3 subcluster carrying NA-I223V is shown with viruses also carrying NA-S247N highlighted. In addition to the subcluster in which the majority of viruses with NA-S247N are allocated (#), in three other branches of subcluster C.5.3.3 NA-I223V the NA-S247N amino acid change occurred (*). Additional amino acid changes that occurred after, or in conjunction with, NA-S247N are shown.

Supplementary Figure S2. Phylogenetic analysis of neuraminidase(NA) sequences of NA-I223V and/or NA-S247N mutant A(H1N1)pdm09 influenza viruses available by 3 August 2026 collected worldwide between October 2023 and April 2026

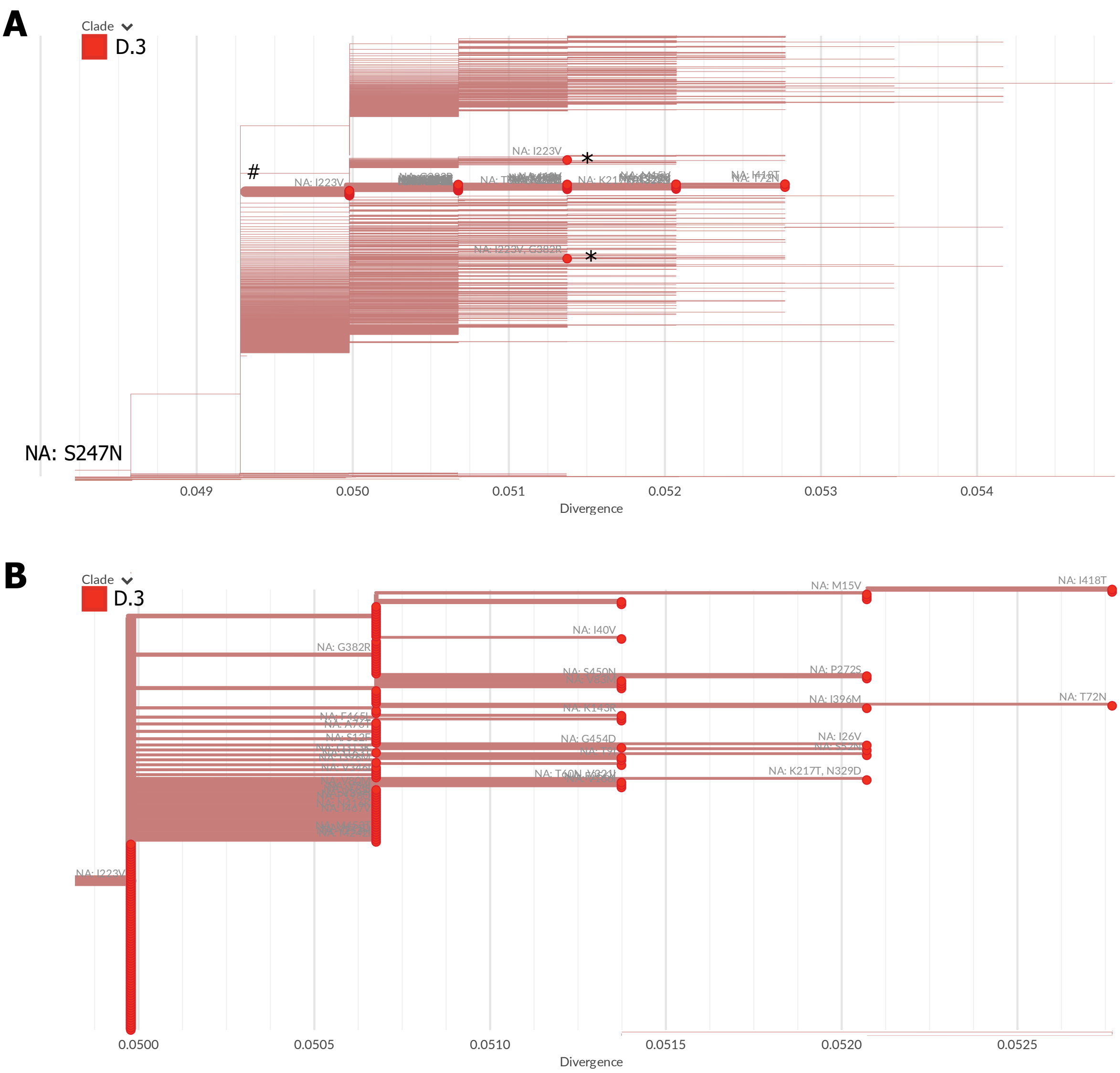

Viruses with NA-S247N and NA-I223V (coloured dots) are placed in the context of viruses that carry NA-I223V or NA-S247N and reference viruses (lines without dots) derived from Nextclade phylogenetic analysis. **A.** The NA-clade D.3 subcluster carrying NA-S247N is shown with viruses also carrying NA-I223V highlighted. In addition to the subcluster in which the majority of viruses with NA-I223V are allocated (#), in two other branches of subcluster D.3 NA-S247N the NA-I223V amino acid change occurred (*). Additional amino acid changes that occurred after, or in conjunction with, NA-I223V are shown. **B.** Zoomed in on the subcluster in which the majority of viruses with NA-I223V are allocated to better show the additional amino acid changes.
